# Contribution of cognitive performance and cognitive decline to associations between socioeconomic factors and dementia: A cohort study

**DOI:** 10.1371/journal.pmed.1002334

**Published:** 2017-06-26

**Authors:** Jennifer Rusmaully, Aline Dugravot, Jean-Paul Moatti, Michael G. Marmot, Alexis Elbaz, Mika Kivimaki, Séverine Sabia, Archana Singh-Manoux

**Affiliations:** 1 INSERM, U1018, Centre for Research in Epidemiology and Population Health, Hôpital Paul Brousse, Villejuif, France; 2 Institut de recherche pour le développement, Marseille, France; 3 Department of Epidemiology and Public Health, University College London, London, United Kingdom; Mayo Clinic, UNITED STATES

## Abstract

**Background:**

Socioeconomic disadvantage is a risk factor for dementia, but longitudinal studies suggest that it does not affect the rate of cognitive decline. Our objective is to understand the manner in which socioeconomic disadvantage shapes dementia risk by examining its associations with midlife cognitive performance and cognitive decline from midlife to old age, including cognitive decline trajectories in those with dementia.

**Methods and findings:**

Data are drawn from the Whitehall II study (*N* = 10,308 at study recruitment in 1985), with cognitive function assessed at 4 waves (1997, 2002, 2007, and 2012). Sociodemographic, behavioural, and cardiometabolic risk factors from 1985 and chronic conditions until the end of follow-up in 2015 (*N* dementia/total = 320/9,938) allowed the use of inverse probability weighting to take into account data missing because of loss to follow-up between the study recruitment in 1985 and the introduction of cognitive tests to the study in 1997. Generalized estimating equations and Cox regression were used to assess associations of socioeconomic markers (height, education, and midlife occupation categorized as low, intermediate, and high to represent hierarchy in the socioeconomic marker) with cognitive performance, cognitive decline, and dementia (*N* dementia/total = 195/7,499). In those with dementia, we examined whether retrospective trajectories of cognitive decline (backward timescale) over 18 years prior to diagnosis differed as a function of socioeconomic markers. Socioeconomic disadvantage was associated with poorer cognitive performance (all *p* < 0.001). Using point estimates for the effect of age, the differences between the high and low socioeconomic groups corresponded to an age effect of 4, 15, and 26 years, for height, education, and midlife occupation, respectively. There was no evidence of faster cognitive decline in socioeconomically disadvantaged groups. Low occupation, but not height or education, was associated with risk of dementia (hazard ratio [HR] = 2.03 [95% confidence interval (CI) 1.23–3.36]) in an analysis adjusted for sociodemographic factors; the excess risk was unchanged after adjustment for cognitive decline but was completely attenuated after adjustment for cognitive performance. In further analyses restricted to those with dementia, retrospective cognitive trajectories over 18 years prior to dementia diagnosis showed faster cognitive decline in the high education (*p* = 0.006) and occupation (*p* = 0.001) groups such that large differences in cognitive performance in midlife were attenuated at dementia diagnosis. A major limitation of our study is the use of electronic health records rather than comprehensive dementia ascertainment.

**Conclusions:**

Our results support the passive or threshold cognitive reserve hypothesis, in that high cognitive reserve is associated with lower risk for dementia because of its association with cognitive performance, which provides a buffer against clinical expression of dementia.

## Abstract 2

In a cohort study including patients from the Whitehall II study, Archana Singh-Manoux and colleagues examine whether cognitive reserve is associated with cognitive performance and rate of cognitive decline.

## Author summary

**Why was this study done?:**

- Socioeconomic disadvantage is associated with greater risk of dementia, but the manner in which socioeconomic factors shape risk of dementia is unclear.
- Previous studies show lower socioeconomic status, a proxy for cognitive reserve, to be associated with lower cognitive function and increased risk of dementia, but not faster cognitive decline.

**What did the researchers do and find?:**

- We used data from 7,499 participants from the Whitehall II study, aged 45–69 years in 1997, who were administered cognitive tests 4 times between 1997 and 2012 and followed for incidence of dementia until 2015.
- We found socioeconomic disadvantage (measured using height, education, and midlife occupational position) to be associated with poorer cognitive performance but not with faster cognitive decline.
- Midlife occupation was associated with risk of dementia, and the excess risk was explained by lower midlife cognitive performance and not faster cognitive decline.
- Analyses restricted to those with dementia showed that the large socioeconomic differences in cognitive performance in midlife were completely attenuated over a period of 18 years leading to dementia diagnosis.

**What do these findings mean?:**

- Our analysis supports the passive or threshold cognitive reserve hypothesis, in that high cognitive reserve is associated with lower risk for dementia because of its association with cognitive performance, which provides a buffer against clinical expression of dementia.
- We found no evidence that high cognitive reserve is associated with a slower rate of cognitive decline; in fact, cognitive decline in the preclinical phase of dementia was more rapid in the high reserve group.
- The main limitation of our study is that analyses are based on dementia ascertained from medical records, likely missing out on milder forms of dementia.

## Introduction

Cognitive reserve refers to an array of factors that provide resilience against neuropathological damage [1–4]. Education and related socioeconomic markers have been found to play such a role [5]. Clinicopathological studies also suggest that although education does not directly affect neurodegeneration or vascular pathologies, it may mitigate the impact of pathology on clinical expression of dementia [6]. Whether this is due to a threshold effect, in that those with more reserve can take more damage before the appearance of clinical symptoms, referred to as the threshold or passive model of reserve, or due to greater efficiency and compensatory mechanisms in those with greater reserve, the active model, remains the subject of debate [1,2,7].

A number of studies show higher risk of dementia in those with lower education [8–10], and recent evidence of a declining prevalence of dementia may be partly attributable to an increase in educational attainment [11–13]. Attempts to understand the role of education have included analysis of cognitive decline using longitudinal data. Early findings showing higher education to be associated with a slow rate of decline [14] have since been shown to have used incorrect statistical methods [15]. More recent studies, with appropriate models of change, suggest education does not influence the age-related rate of cognitive decline [16–20]. All these studies show large differences in cognitive performance; for example, a recent study concluded that at age 55 years the difference in cognitive performance between groups with less than high school education and those with a university or equivalent degree corresponded to an age effect of 22 years [19]. Whether these differences translate into differences in age of onset of dementia or rate of cognitive decline prior to dementia remains unknown.

In the population-based Whitehall II study, we sought to determine the importance of the threshold and active models of reserve using markers of socioeconomic circumstances as proxy measures of cognitive reserve. Height, a marker of early socioeconomic circumstances; education; and midlife occupation represented socioeconomic circumstances through the life course in order to allow an evaluation of the salience of critical exposure periods for cognitive outcomes. Longitudinal data on cognitive trajectories along with data on dementia were used to examine the manner in which cognitive reserve shapes cognitive ageing. The threshold model of reserve would suggest that socioeconomic position is associated primarily with cognitive performance but not decline and that its association with risk of dementia is due to the buffer provided by higher cognitive performance in socioeconomically advantaged groups. The active model of reserve would be supported if socioeconomic advantage was associated with slower age-related cognitive decline.

Given the lack of associations between socioeconomic markers and cognitive decline reported in recent studies [16–20], we hypothesized that the association between socioeconomic factors and dementia would be explained by cognitive performance, thus supporting the threshold model of reserve. In further analyses, focused on those with dementia in order to assess the manner in which cognitive reserve shapes cognitive trajectories in this group, we examined whether socioeconomic markers were associated with cognitive decline preceding dementia diagnosis. Assessment of these retrospective cognitive trajectories will allow us to determine whether large differences in cognitive performance in midlife attributed to cognitive reserve, estimated to correspond to an age effect of over 20 years [19], change in the preclinical period of dementia.

## Methods

### Study design and participants

**Fig 1:**
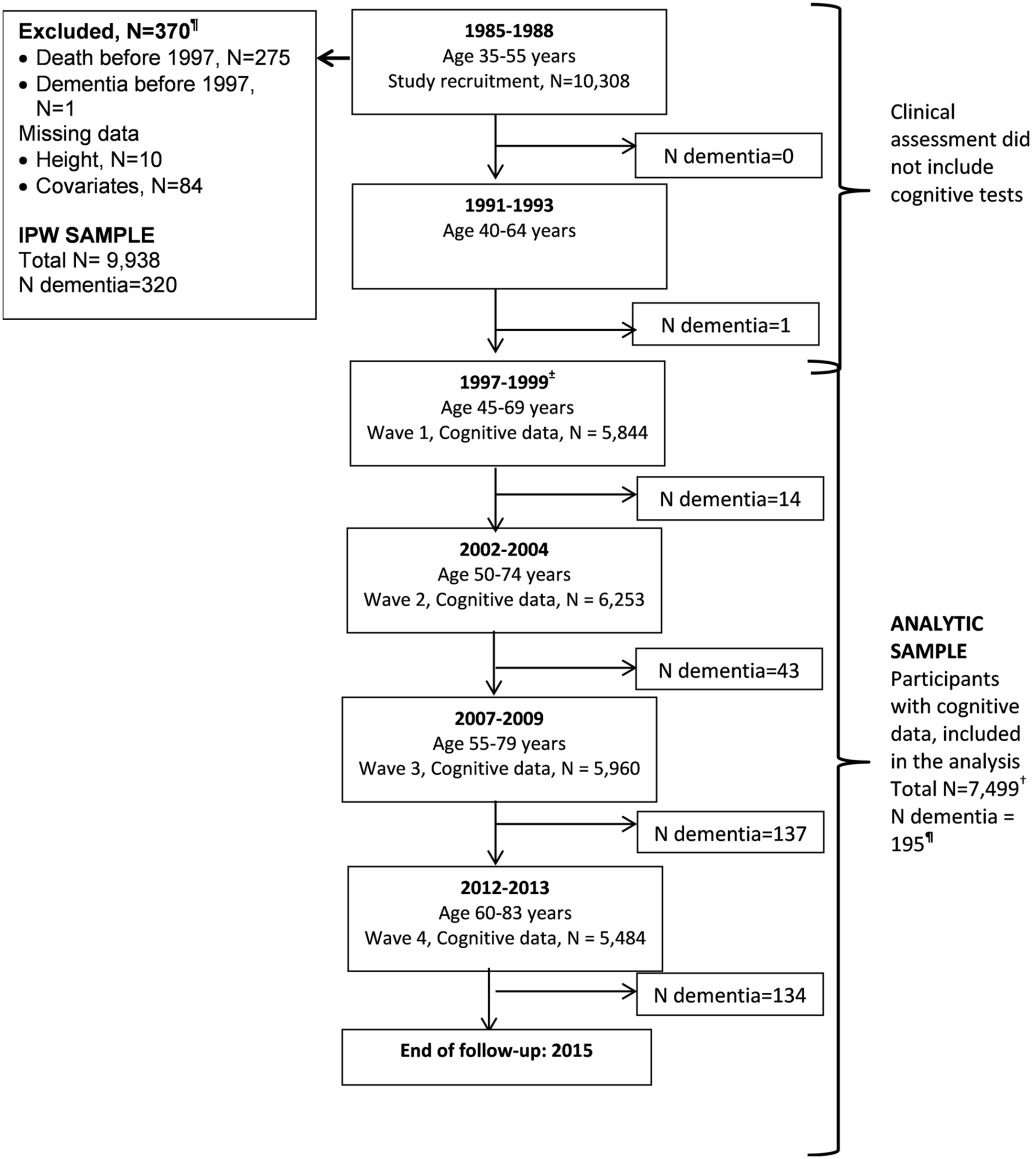
Flow chart for study of cognitive decline and dementia. Abbreviations: IPW, inverse probability weighting. ^**¶**^Of the 329 participants with dementia recorded until 31 March 2015, 9 were in those with data missing on covariates or onset of dementia was prior to 1997, allowing 320 participants to be included in IPW. In those with cognitive data (*N* = 7,499), a total of 195 participants had a dementia diagnosis over the follow-up. ^**±**^ Cognitive tests were introduced to the study at the 1997–1999 assessment, repeated at subsequent assessments. ^†^ A total of 7,499 participants were included in the analysis; 43% had cognitive data at all 4 waves, 28% at 3 waves, 15% at 2 waves, and 14% only at 1 wave.

The Whitehall II study is an ongoing cohort study of men and women on 10,308 persons (6,895 men and 3,413 women) working in British Civil Service departments, aged 35–55 years, recruited to the study in 1985 [21]. All participants responded to a questionnaire and underwent a structured clinical evaluation, consisting of measures of anthropometry, cardiovascular and metabolic risk factors, and disease. Since the baseline medical examination, follow-up examinations have taken place approximately every 5 years (flow chart, Fig 1). A battery of cognitive tests was introduced to the study in 1997–1999, making it the baseline of our analysis. Written consent from the participant and research ethics approvals (University College London [UCL] ethics committee) are renewed at each contact; the latest approval was by the Joint UCL/UCLH Committee on the Ethics of Human Research (Committee Alpha), reference number 85/0938.

### Measures

#### Socioeconomic factors (1997)

Height was measured using a stadiometer, with the participant standing completely erect with his or her head in the Frankfort plane. Tertiles of height were used: 174–179 cm (160–165 cm) in men (women) was the middle category, and height above or below this range constituted the other 2 categories. Education was measured as the highest qualification on leaving full-time education and categorised as lower secondary school or less, higher secondary school, or university or higher degree. Occupational position was assessed by the British Civil Service grade of employment, a 3-level variable representing high (administrative grades), intermediate (professional or executive grades), and low (clerical or support grades) position. This measure is a marker of socioeconomic circumstances and is related to salary, social status, and level of responsibility at work in the British Civil Service [21]. The categories of these socioeconomic markers (low, intermediate, and high) reflect the social gradient, allowing putative dose-response associations to be estimated.

#### Global cognitive score

The cognitive test battery was used at assessments in 1997, 2003, 2007, and 2012 [22]. It consisted of tests of memory, assessed using a 20-word free recall test; reasoning, assessed via the Alice Heim 4-I test composed of a series of 65 verbal and mathematical reasoning items of increasing difficulty [23]; and verbal fluency, using measures of phonemic (words starting with *s*) and semantic (animal names) fluency [24]. A global cognitive score was created using all the tests described above by first standardizing the raw scores on each test to z-scores using mean and standard deviation (SD) from the 1997 wave [22]. The z-scores were then averaged and standardized to yield the global cognitive score.

#### Dementia

We used comprehensive tracing of electronic health records for dementia ascertainment using 3 databases: the national hospital episode statistics (HES) database, the Mental Health Services Data Set (MHSDS), and the mortality register. Record linkage until 31 March 2015, using International Classification of Diseases Tenth Edition (ICD-10) codes F00, F01, F02, F03, F05.1, G30, G31.0, G31.1, and G31.8, to identify participants with dementia, following the National Health Service (NHS) guidelines. In the United Kingdom (England, Scotland, and Wales), the NHS provides most of the health care, including out- and in-patient care. MHSDS is a national database that contains information for persons in contact with mental health services in hospitals, outpatient clinics, and the community.

#### Covariates

Sociodemographic factors included age, sex, ethnicity (white and nonwhite), and marital status (married/cohabiting versus other). Health behaviours included smoking (current, ex-, and never smokers), alcohol consumption (number of alcoholic drinks consumed in the last 7 days, converted to units and categorized as no/occasional, moderate [1–14 units/week in women and 1–21 units/week in men], and heavy alcohol consumption [≥14 units in women and ≥21 units in men]), physical activity (“active”: ≥2.5 hours/week of moderate physical activity or ≥1 hour/week of vigorous physical activity; “inactive”: <1 hour/week of moderate and vigorous activity; and intermediate level of activity for all others), and frequency of fruit and vegetable consumed per week (categorized as less than once/day, once/day, and more often).

Health status covariates included hypertension (systolic/diastolic ≥ 140/90 mmHg or use of antihypertensive medication), prevalent diabetes mellitus (determined by fasting glucose ≥ 7.0 mmol/l, a 2-hour postload glucose ≥ 11.1 mmol/l, reported doctor-diagnosed diabetes, or use of diabetes medication), self-reported use of medication for cardiovascular disease, mental health (30-item General Health Questionnaire) [25], and HES linkage data on coronary heart disease (CHD; ICD codes: I20–I25), stroke (ICD codes: I60–I64), and chronic obstructive pulmonary disease (ICD codes: J41–J44).

### Statistical analysis

All analyses were based on 7,499 participants (flow chart, Fig 1) because of loss to follow-up between the study recruitment in 1985 (*N* = 10,308) and the introduction of cognitive tests to the study protocol in 1997. We examined associations between participant characteristics at the beginning (1997–1999) of the cognitive follow-up using χ^2^ tests (categorical data) and analysis of variance (continuous data).

Analyses were weighted to take into account missing data using inverse probability weighting (IPW) [26]. In order to do this, we used data on 9,938 participants who in 1997 (start of cognitive testing) were alive and nondemented and had data on socioeconomic markers. The probability of remaining in the study sample was estimated using data on sociodemographic measures (age, sex, ethnicity, education, height, occupational position, and marital status), health behaviours (smoking, alcohol consumption, physical activity, and fruit and vegetable consumption), cardiometabolic risk factors (body mass index, systolic and diastolic blood pressure, and cholesterol), and mental health (General Health Questionnaire) from the study baseline (1985) and chronic conditions (CHD, stroke, diabetes, chronic obstructive pulmonary disease, cancer), including dementia status, over the follow-up (1985 to 2015). We also included interaction terms between dementia status and height, education, and occupation in the calculation of weights. The inverse of these probabilities was used to weight the analyses.

#### Analysis of cognitive performance and decline

The association of socioeconomic markers with cognitive performance and cognitive decline (using 4 waves of data) was examined using a weighted generalized estimating equations (GEE) linear regression model with an unstructured correlation structure [27]. For these analyses, weights were calculated for each individual, for each wave of cognitive data, based on those who were alive at that wave. These models use all available data over the follow-up and take into account the fact that repeated measures on the same individual are correlated. We used GEE models rather than linear mixed effects models in order to incorporate IPW in the analyses. The model contained time, time squared, age in 1997–1999, sex, ethnicity, their interactions with time and time squared, and time-dependant marital status. Subsequent analyses were further adjusted for health behaviours and health status, treated as time-dependent covariates.

*Sensitivity analysis*: Our main analysis using weighted GEE is based on a missing at random (MAR) assumption, i.e., given the variables used to define weights, data are missing independently of unobserved data. As this assumption is untestable, we undertook sensitivity analyses under a missing not at random (MNAR) assumption following 3 steps. First, we used multiple imputation (20 datasets) to impute missing cognitive data based on covariates used in IPW and all cognitive data, measures of functional status using the SF36 physical and mental component scores, and instrumental and basic activities of daily living (IADL and ADL) over the follow-up (1997 to 2012); data imputed after death were set as missing. Second, we replaced the imputed cognitive data by values that were 0.2 or 0.5 SD lower, i.e., those who had missing cognitive data are hypothesized to systematically have lower scores than participants with the same characteristics for whom cognitive data were available. Third, we repeated the weighted GEE analysis on the imputed dataset using Rubin’s rule to compute estimates.

#### Analysis of incident dementia

Follow-up started at the 1997–1999 assessment, and participants were censored at date of record of dementia, death (to account for competing risk of mortality), or 31 March 2015, whichever came first. As there was no evidence of sex differences in associations (*p* for interaction for height = 0.09, education = 0.40, and occupation = 0.86), men and women were combined in Cox proportional hazards models, with age as the timescale.

The first set of analyses examined the role of health behaviours and health status in explaining the association between socioeconomic markers and dementia by entering them as time-varying covariates in the model. We then examined whether baseline cognitive performance or cognitive decline explained the association between socioeconomic markers and dementia. This was done by first deriving person-specific random effects from a mixed-effects model (random slope and intercept) and then entering the random intercept in the Cox regression to assess the importance of cognitive performance and repeating it with the random slope to assess the importance of cognitive decline.

#### Retrospective analysis of cognitive trajectory in those with dementia

In those with a dementia diagnosis who also had cognitive data (*N* = 195), we examined whether their cognitive decline trajectory varied as a function of height, education, or occupation. A backward timescale was used, such that Year = 0 was the year of dementia diagnosis, and cognitive decline trajectories over 18 years prior to Year 0 were modelled using mixed effects models, with random intercept and slope [28]. Differences in trajectory of the global cognitive score between participants with dementia in the different socioeconomic groups were tested by assessing whether the interaction between socioeconomic categories and slope terms (time and time^2^) improved fit of the model using the likelihood ratio test. Analyses were adjusted for age at onset of dementia, sex, ethnicity, their interactions with time, and for time squared, 5-year birth cohort (to take cohort effects into account), and time-dependant marital status.

## Results

The 7,499 participants (Fig 1) included in our analysis compared to 9,938 participants used in IPW were more likely to be younger, male, from the socioeconomically advantaged group, and have a better health profile, including a lower dementia rate (S1 Table). Table 1 presents sample characteristics of those included in the analyses at the beginning of cognitive testing in 1997.

**Table 1:** Characteristics of the study population (*N* = 7,499 at study baseline [1997], beginning of cognitive testing).

	Total	No dementia	Dementia	
	7,499	7,304	195	*p*
Female, %	29.68	29.53	35.38	0.077
Age, M (SD)	55.63 (5.97)	55.47 (5.93)	61.82 (4.17)	<0.001
Nonwhite, %	8.64	8.54	12.31	0.065
Single, %	23.64	23.69	22.05	0.596
Height (metres), M (SD)	1.72 (0.09)	1.72 (0.09)	1.71 (0.10)	0.040
Low education, %	44.27	43.99	54.87	0.003
Low occupational position, %	13.82	13.51	25.13	<0.001
Physically inactive^†^, %	17.07	16.91	23.08	0.024
Poor diet^±^, %	27.88	27.90	27.18	0.824
Nonmoderate alcohol consumption^‡^, %	39.51	39.27	48.72	<0.008
Current smokers, %	10.45	10.50	8.72	0.422
Diabetes, %	4.83	4.27	10.26	<0.001
Hypertension, %	28.10	27.90	35.90	0.014
COPD, %	0.03	0.03	0.00	0.817
Cardiovascular disease, %	5.53	5.49	7.18	0.309
CVD medication, %	17.70	17.47	26.15	0.002
GHQ score, M (SD)	3.13 (5.53)	3.14 (5.54)	2.78 (5.10)	0.376

^**†**^Corresponds to <1 hour/week of moderately energetic activity and <1 hour/week of vigorous physical activity.

^**±**^Corresponds to fruit and vegetable consumption < once a day.

^**‡**^Other than 1–14 units/week in women and 1–21 units/week in men.

Abbreviations: COPD, chronic obstructive pulmonary disease; CVD, cardiovascular disease, including coronary heart disease and stroke; GHQ, General Health Questionnaire; M, mean; SD, standard deviation.

The associations of socioeconomic markers with cognitive performance and cognitive decline, analysed using weighted GEE models and adjusted for sociodemographic covariates, are shown in Table 2. For cognitive performance (left panel), the associations were weakest with height and strongest with occupation. To allow interpretation of regression coefficients, we compared them with the effect of age on cognition by dividing the estimate by the effect of a 1-year increase in age on cognitive performance, calculated by regressing the standardised global cognitive score in 1997 on age (beta = −0.0496). The difference in cognitive performance in the low versus the high height group corresponded to an age effect of 4.2 years; the corresponding effects in low education and occupation groups were 15.1 and 25.8 years using point estimates for the effect of age.

**Table 2:** Association of height, education, and occupation with performance and decline in the global cognitive score^†^.

	Cognitive Performance (1997)			15-Year Cognitive Decline (1997–2012)		
	Mean (95% CI)	Difference (95% CI)	*p*	Mean (95% CI)	Difference (95% CI)	*p*
**Height**						
High	0.05 (0.02 to 0.09)	Reference		-0.63 (-0.66 to -0.60)	Reference	
Intermediate	−0.07 (−0.11 to −0.03)	−0.12 (−0.18 to −0.07)	<0.001	−0.61 (−0.64 to −0.58)	0.02 (−0.02 to 0.07)	0.286
Low	−0.16 (−0.20 to −0.12)	−0.21 (−0.27 to −0.16)	<0.001	−0.63 (−0.67 to −0.60)	0.00 (−0.05 to 0.05)	0.967
**Education**						
High	0.38 (0.34 to 0.41)	Reference		−0.65 (−0.69 to −0.62)	Reference	
Intermediate	0.03 (−0.01 to 0.07)	−0.35 (−0.40 to −0.29)	<0.001	−0.63 (−0.67 to −0.60)	0.02 (−0.03 to 0.07)	0.384
Low	−0.37 (−0.40 to −0.34)	−0.75 (−0.80 to −0.69)	<0.001	−0.61 (−0.64 to −0.58)	0.04 (−0.00 to 0.09)	0.073
**Occupation**						
High	0.39 (0.36 to 0.42)	Reference		−0.65 (−0.68 to −0.62)	Reference	
Intermediate	−0.18 (−0.21 to −0.15)	−0.56 (−0.61 to −0.52)	<0.001	−0.63 (−0.66 to −0.61)	0.01 (−0.03 to 0.05)	0.524
Low	−0.89 (−0.96 to −0.82)	−1.28 (−1.36 to −1.20)	<0.001	−0.61 (−0.67 to −0.55)	0.04 (−0.03 to 0.11)	0.307

^**†**^Inversely probability-weighted generalized-estimating-equation models adjusted for age, sex, ethnicity, and time-dependant marital status. Abbreviations: CI, confidence interval.

The right panel of Table 2 shows no association between socioeconomic markers and cognitive decline. In order to assess whether dementia occurring over the follow-up affected these findings, we repeated these analyses after removing all participants with dementia. These results (S2 Table) show further attenuation in differences in cognitive decline between the high and low education and occupation groups. Adjustment for health behaviours and health status did not significantly change the associations of socioeconomic markers with cognitive performance and cognitive decline (S3 Table).

Sensitivity analyses, undertaken to assess whether results would be similar under a MNAR assumption, estimated the association of socioeconomic markers with cognitive performance and cognitive decline. These results show that the 2 scenarios of nonresponse—persons with missing data had 0.2 SD and 0.5 SD lower scores—produce associations with cognitive performance (S4 Table) and cognitive decline (S5 Table) that were similar to those observed in the IPW analyses (Table 2). These results support the adequacy of the IPW procedure in our analysis.

A total of 329 participants with dementia were idenitified up to the 31 March 2015 (Fig 1). One occurred before 1997, and covariate data were missing for 8 participants, leaving 320 participants with dementia in the analysis over a mean follow-up of 16.1 years. The mean age of participants at dementia diagnosis was 75.6 (SD = 4.6) years and was similar in the high, intermediate, and low groups defined by height (75.1, 76.1, and 75.6 years), education (75.5, 74.2, and 76.3 years), and occupation (75.2, 75.4, and 76.6 years). In unadjusted analyses (S6 Table), small stature (hazard ratio [HR] = 1.46; 95% confidence interval [CI] 1.11–1.91), low education (HR = 1.85; 95% CI 1.39–2.46), and occupation (HR = 2.77; 95% CI 2.11–3.65) were all associated with higher risk of dementia. However, adjustment for sociodemographic factors, mainly age, (Model 1, Table 3) left only an association with occupation. The first set of results in Table 3 (Model 1, without IPW) included 320 participants with dementia, and the IPW models, restricted to those on whom we have cognitive data (*N* = 7,499), included 195 participants with dementia. Similar results in both sets of analysis suggest adequacy of the weights used. In IPW analysis adjusted for sociodemographic covariates, the hazard of dementia was 2.03 (95% CI 1.23–3.36) times higher in the low compared to the high occupation category. Adjustment for health behaviours and health status attenuated this association (HR = 1.55; 95% CI 0.93–2.58).

**Table 3:** Association of height, education, and occupation with dementia: Role of health behaviours and health status.

	Dementia study population		IPW analyses (on those with cognitive data)				
	*N* Dementia / Total = 320/9,938		*N* Dementia / Total = 195/7,499				
	*N* dementia / *N* total	Model 1 (without IPW)	*N* dementia / *N* total	Model 1	Model 1 + health behaviours^±^	Model 1 + Health status^≠^	Model 1 + health behaviours and health status
		HR (95% CI)		HR (95% CI)	HR (95% CI)	HR (95% CI)	HR (95% CI)
**HEIGHT**							
High	89/3,261	Reference	52/2,567	Reference	Reference	Reference	Reference
Intermediate	103/3,364	1.00 (0.75–1.33)	72/2,558	1.12 (0.78–1.63)	1.07 (0.73, 1.56)	1.09 (0.76–1.57)	1.06 (0.73–1.54)
Low	128/3,313	1.16 (0.88–1.54)	71/2,374	1.13 (0.77–1.67)	1.06 (0.71–1.57)	1.14 (0.77–1.69)	1.08 (0.73–1.62)
**EDUCATION**							
High	61/2,603	Reference	44/2,171	Reference	Reference	Reference	Reference
Intermediate	65/2,639	0.96 (0.68–1.36)	44/2,008	1.00 (0.64–1.57)	0.99 (0.63–1.57)	1.01 (0.64–1.59)	1.00 (0.63–1.58)
Low	194/4,696	1.02 (0.75–1.38)	107/3,320	1.05 (0.72–1.55)	0.98 (0.66–1.45)	1.03 (0.70–1.53)	0.99 (0.66–1.48)
**OCCUPATION**							
High	89/3,705	Reference	69/3,158	Reference	Reference	Reference	Reference
Intermediate	111/4,319	1.13 (0.84–1.51)	77/3,305	1.22 (0.87–1.73)	1.13 (0.79–1.61)	1.15 (0.81–1.62)	1.08 (0.76–1.54)
Low	120/1,914	2.06 (1.46–2.91)^c^	49/1,036	2.03 (1.23–3.36)^b^	1.64 (0.95–2.82)	1.77 (1.10–2.84)^a^	1.55 (0.93–2.58)

^a^*p* < 0.05,

^b^*p* < 0.01,

^c^*p* < 0.001.

^±^Smoking, alcohol consumption, physical activity, and fruit and vegetable consumption.

^≠^Hypertension, diabetes, use of medication for cardiovascular disease, anxiety and depression symptoms, cardiovascular disease, and chronic obstructive pulmonary disease.

Model 1: Cox model with age as the timescale and adjusted for demographic characteristics (sex, ethnicity, and time-dependant marital status) and 5-year birth cohort. Abbreviations: CI, confidence interval; HR, hazard ratio; IPW, inverse probability weighting.

In analysis adjusted for sociodemographic measures, both better cognitive performance (HR per 1 SD increment = 0.55; 95% CI 0.47–0.65) and slower cognitive decline (HR per 1 SD slower decline = 0.80; 95% CI 0.70–0.90) were associated with lower hazard of dementia (S7 Table). Table 4 shows the results of the analysis aimed at determining whether the association of socioeconomic markers with dementia was explained by cognitive performance or cognitive decline. For occupation (the only marker associated with dementia), excess risk was attenuated after adjustment for cognitive performance (HR in low versus high group = 0.89; 95% CI 0.50–1.58) but not cognitive decline (HR in low versus high group = 2.10; 95% CI 1.27–3.48).

**Table 4:** Association of height, education, and occupation with dementia: Role of cognitive performance and cognitive decline.

IPW analyses: On those with cognitive data					
*N* Dementia / Total = 195/7,499					
	*N* dementia / *N* total	Model 1	Model 1 + cognitive performance	Model 1 + 15-year cognitive decline	Model 1 + cognitive performance and 15-year cognitive decline
		HR (95% CI)	HR (95% CI)	HR (95% CI)	HR (95% CI)
**HEIGHT**					
High	52/2,567	Reference	Reference	Reference	Reference
Intermediate	72/2,558	1.12 (0.78–1.63)	1.05 (0.73–1.53)	1.11 (0.75–1.65)	1.05 (0.72–1.51)
Low	71/2,374	1.13 (0.77–1.67)	0.98 (0.66–1.45)	1.13 (0.78–1.63)	0.96 (0.64–1.42)
**EDUCATION**					
High	44/2,171	Reference	Reference	Reference	Reference
Intermediate	44/2,008	1.00 (0.64–1.57)	0.82 (0.52–1.30)	1.02 (0.65–1.60)	0.83 (0.53–1.32)
Low	107/3,320	1.05 (0.72–1.55)	0.68 (0.44–1.03)	1.08 (0.74–1.58)	0.67 (0.44–1.01)
**OCCUPATION**					
High	69/3,158	Reference	Reference	Reference	Reference
Intermediate	77/3,305	1.22 (0.87–1.73)	0.85 (0.59–1.23)	1.25 (0.89–1.75)	0.82 (0.57–1.19)
Low	49/1,036	2.03 (1.23–3.36)^a^	0.89 (0.50–1.58)	2.10 (1.27–3.48)^a^	0.83 (0.47–1.48)

^a^*p* < 0.01

Model 1: Cox model with age as timescale and adjusted for demographic characteristics (sex, ethnicity, and time-dependant marital status) and 5-year birth cohort. Abbreviations: CI, confidence interval; HR, hazard ratio; IPW, inverse probability weighting.

**Fig 2:**
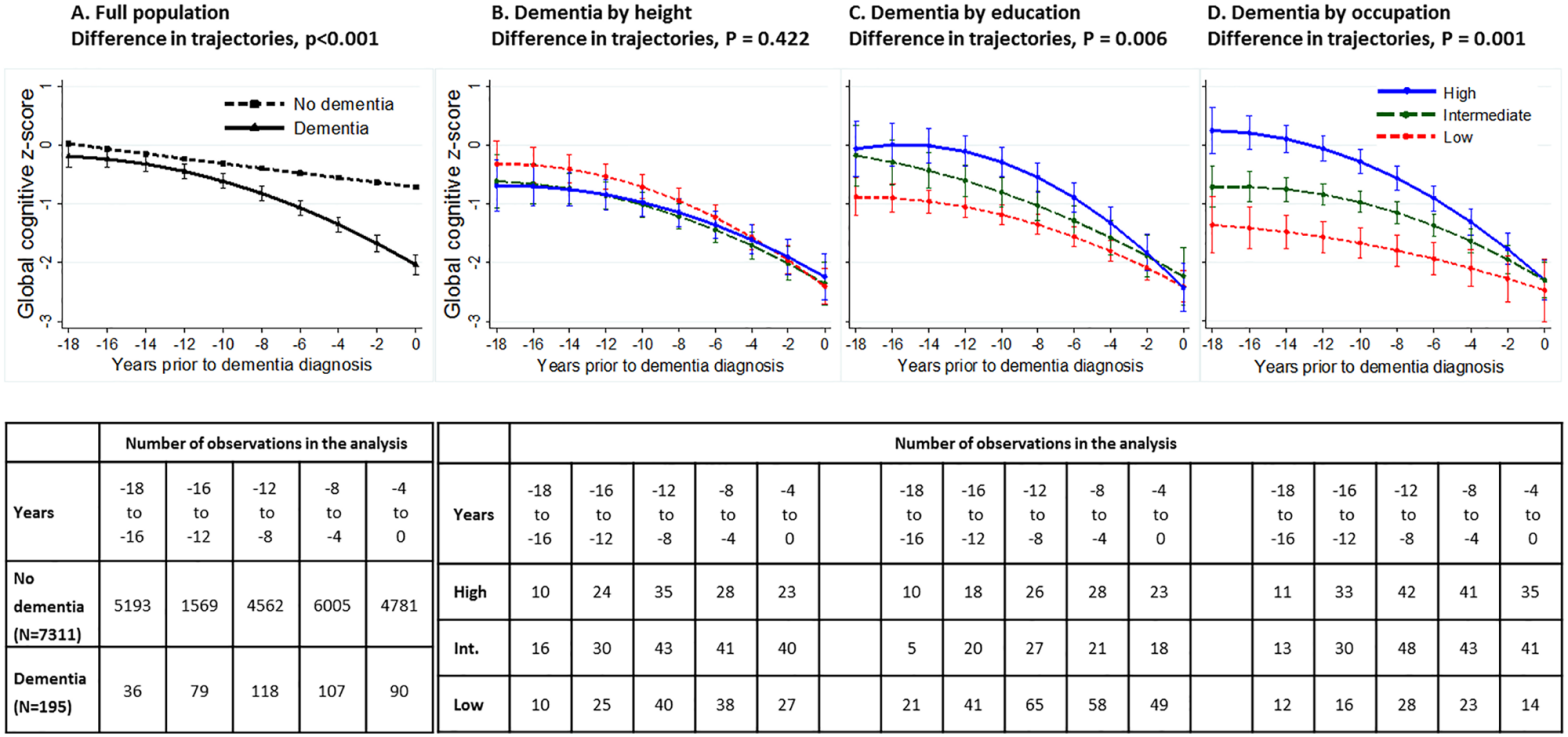
Trajectory of global cognitive score in participants with dementia and other participants in the years leading to dementia diagnosis in the total population (panel A) [29] and in those with dementia as a function of height (panel B), education (panel C), and occupation (panel D). ^† †^Panel A depicts marginal effects of dementia on trajectories of the global cognitive score (composed of tests of memory, reasoning, and phonemic and semantic fluency), adjusted for age at the end of follow-up (dementia diagnosis, death, or 31 March 2015), 5-year birth cohort, sex, and education. Results for height, education, and occupation include age at onset of dementia, sex, ethnicity, their interactions with time and time squared, 5-year birth cohort to take cohort effects into account, and time-dependant marital status.

Fig 2 (panel A) [29] characterises changes in the global cognitive score over 18 years prior to dementia diagnosis, showing accelerated decline in the 8–10 years before dementia diagnosis, as has been shown in studies that use a “gold-standard” dementia ascertainment procedure [28]. Whether height (panel B), education (panel C), or occupation (panel D) affected cognitive trajectories in analysis restricted to those with dementia is also shown in Fig 2. Education (*p* = 0.006) and occupation (*p* = 0.001), but not height (*p* = 0.422), affected trajectories. The high occupation group had a 1.61 SD (*p* < 0.001) higher cognitive score than the low occupation group 18 years prior to dementia diagnosis, but these differences were attenuated by the year of dementia diagnosis (S8 Table), showing faster cognitive decline in the high compared to the low socioeconomic group in the years leading up to dementia diagnosis.

## Discussion

In this population-based, longitudinal study of persons aged 45 to 69 years at the first cognitive assessment, socioeconomic disadvantage was associated with poorer cognitive performance but not faster cognitive decline. The associations with cognitive performance were strongest for occupation and weakest for height. After accounting for sociodemographic measures, the increased risk of dementia was evident only for low occupation. The analysis of cognitive trajectories before the diagnosis of dementia showed much higher cognitive performance in the high compared to low occupational groups 18 years before diagnosis, but this difference was completely attenuated at dementia diagnosis due to faster cognitive decline in the high occupation group in the years preceding dementia diagnosis.

Use of 3 socioeconomic markers over the life course allowed us to assess whether the timing of exposure matters for cognitive outcomes. In our cohort of participants born between 1930 and 1952, height is probably a better marker of early socioeconomic circumstances than in more recent birth cohorts. Our data show that height had the weakest association with cognitive performance. Data from other industrialized countries on older adults also show an association between height and cognitive function [30]; in our study, there was no evidence of an association with cognitive decline or dementia. Of the 3 socioeconomic markers we examined, education has generated the most interest in previous studies on dementia. As suggested by a recent review [31], this association is susceptible to cohort effects in that associations with dementia are not always found in younger cohorts. In our data, low education was associated with increased hazard of dementia in models unadjusted for age; inclusion of age removed the excess risk.

Occupation had a robust association with dementia, and health behaviours explained a large part of this excess risk. These findings emphasize the importance of a healthy lifestyle for dementia prevention. The importance of occupation for cognitive performance could also be due to the impact of environments encountered after the completion of education that induce practice and reinforcement of cognitive abilities. Findings from animal studies show enriched environments to improve brain structure and function [32,33]. However, our findings could also be explained by social selection, processes that lead individual differences in cognitive functioning to shape social mobility [34]. The 2 explanations are not mutually exclusive and may operate at different times across the life course.

Our findings on cognitive decline are in agreement with recent studies that show socioeconomic disadvantage not to be associated with a faster rate of cognitive decline [16–20]. Indeed, several studies have reported faster cognitive decline in high compared to low education and occupation groups [16,35]. These findings might reflect the transience of cognitive gains associated with education or work content, which are lost at older ages. Other explanations involve statistical phenomena such as regression to the mean [36] and floor or ceiling effects. A recent paper examined several explanations for slower cognitive decline in the low education group and found differences between education groups to become nonsignificant once selective attrition, due to nonresponse and death, was taken into account [35]. We used a similar method to account for attrition and found the rates of cognitive decline not to be associated with socioeconomic position. Additional analyses in our study suggest that faster cognitive decline in those who develop dementia in the high education/occupation group is another explanation: removing all participants with dementia over the follow-up further reduced differences in cognitive decline (S2 Table).

There is considerable interest in predictors of cognitive decline, as they may be amenable to modification, facilitating prevention of progression to cognitive impairment or dementia. In observational data, the associations of putative risk factors with cognitive decline compared to cognitive performance are less biased by confounding, as these factors are likely to be stable over time within individuals. In the context of the cognitive reserve hypothesis, the relative importance of cognitive performance and cognitive decline has generated much research interest within the framework of the cognitive reserve hypothesis [1,2]. Our study supports the importance of cognitive performance; its inclusion completely attenuated the excess risk of dementia in the low occupation group, supporting the threshold model of reserve. No such effects were seen for cognitive decline, suggesting that cognitive reserve influences dementia risk primarily by boosting cognitive performance. These findings have implications for interventions based on cognitive stimulation at older ages that attempt to prevent dementia. In line with our results, there is little support for the hypothesis that mental [37] or cognitive training [38] reduces the rate of cognitive decline.

It has been suggested that education does not affect neurodegeneration or vascular pathologies in the brain but may affect its clinical expression [5,6]. Our analysis of cognitive trajectories over 18 years, modelled backwards, in those with dementia supports this view. Most dementias unfold over a long period [39], perhaps more than 2 decades [40]. In the 18-year retrospective observation window in our study, those with dementia in the high education and occupation groups experienced more rapid decline so that differences in cognitive performance were attenuated at dementia diagnosis. Although the number of participants with dementia in these analyses is relatively small, it is unlikely that dementia diagnosis differs much as a function of socioeconomic markers. If this were the case, the age of dementia diagnosis would not be similar across the socioeconomic groups. Considered together, our results do not provide any evidence that high cognitive reserve is associated with slower cognitive decline.

The main strengths of this study include the large sample size and consideration of cognitive performance, decline, and dementia using longitudinal data to study cognitive reserve. We used a battery of cognitive tests and present results using the global cognitive score, allowing replication across studies in the future. A further strength is the use of IPW to take into account biases that arise because of missing data. Availability of data at study recruitment and over the follow-up allowed us to incorporate this information into the construction of weights. The adequacy of the IPW procedure was supported by the comparison of the associations between socioeconomic markers and dementia in the larger sample and the analytic sample. The person- and wave- specific weights used in the analysis of cognitive trajectories, including data on dementia and chronic diseases over the follow-up, allowed us to reduce bias due to missing data. Sensitivity analyses under the MNAR assumption also suggest that our results are unlikely to be influenced by missing data.

Limitations to this study warrant consideration. The Whitehall II study was an occupational cohort at recruitment and therefore not representative of the UK population. We have previously shown the risk factor—CHD associations in our study to be similar to those in a general population study [41], suggesting that the present findings are likely to be generalizable. Another limitation of the study is ascertainment of dementia being based on linkage to electronic health records. While the specificity of diagnosis with this method is likely to be high, the sensitivity is undoubtedly low. As undiagnosed dementia becomes common only in very old age, underascertainment is unlikely to unduly affect our findings. The advantage of our method of ascertainment is that it is independent of the Whitehall II study and less affected by ascertainment biases that can occur in population-based studies that use poor performance to screen for further testing for dementia.

The salience of our study is tied to the view that various forms of neurodegeneration are increasingly seen as a continuum, with a lack of clear distinction between normal ageing, abnormal cognitive decline, and dementia, both in symptoms and histopathology [42,43]. Thus, consideration of cognitive performance, decline, and dementia allows a comprehensive assessment of the importance of putative risk factors. Our results have 2 implications. One, the association was strongest with occupation, suggesting considerable plasticity in functioning up to at least midlife. Two, none of the markers of socioeconomic advantage were associated with slower cognitive decline, suggesting that enriched environments affect cognitive performance rather than prevent loss in cognitive function, supporting the threshold model of reserve. The precise nature of these environments is important to determine in order to forestall the clinical manifestation of dementia.

## Supporting information

S1 Table Sample characteristics of participants included and not included in the analysis—Overall and as a function of dementia status. (DOCX)

S2 Table Association of height, education, and occupation with performance and decline in the global cognitive score, excluding participants with dementia. (DOCX)

S3 Table Association of height, education, and occupation with performance and decline in the global cognitive score: Adjustment for health behaviours and health status. (DOCX)

S4 Table Sensitivity analysis: Association of socioeconomic markers with cognitive performance; missing not at random (MNAR) assumption. (DOCX)

S5 Table Sensitivity analysis: Association of socioeconomic markers with cognitive decline; missing not at random (MNAR) assumption. (DOCX)

S6 Table Association of height, education, and occupation with dementia: Unadjusted analyses. (DOCX)

S7 Table Association of cognitive performance and cognitive decline with dementia. (DOCX)

S8 Table Difference in cognitive trajectories prior to dementia by socioeconomic indicators. (DOCX)

S1 Text Prospective analysis plan. (DOCX)

S2 Text Strengthening the Reporting of Observational Studies in Epidemiology (STROBE) Statement—Checklist of items that should be included in reports of observational studies. (DOCX)

## Abbreviations

CHD: coronary heart disease
CI: confidence interval
COPD: chronic obstructive pulmonary disease
CVD: cardiovascular disease, including coronary heart disease and stroke
GHQ: General Health Questionnaire
HR: hazard ratio
IPW: inverse probability weighting
M: mean
MNAR: missing not at random
SD: standard deviation
STROBE: Strengthening the Reporting of Observational Studies in Epidemiology

## References

[1] Stern Y. What is cognitive reserve? Theory and research application of the reserve concept. J Int Neuropsychol Soc. 2002;8(3):448–60. 11939702

[2] Katzman R. Education and the prevalence of dementia and Alzheimer's disease. Neurology. 1993;43(1):13–20. 8423876 10.1212/wnl.43.1_part_1.13

[3] Price JL, Morris JC. Tangles and plaques in nondemented aging and "preclinical" Alzheimer's disease. Ann Neurol. 1999;45(3):358–68. 10072051 10.1002/1531-8249(199903)45:3<358::aid-ana12>3.0.co;2-x

[4] Crystal H, Dickson D, Fuld P, Masur D, Scott R, Mehler M, et al Clinico-pathologic studies in dementia: nondemented subjects with pathologically confirmed Alzheimer's disease. Neurology. 1988;38(11):1682–7. 3185902 10.1212/wnl.38.11.1682

[5] Bennett DA, Wilson RS, Schneider JA, Evans DA, Mendes de Leon CF, Arnold SE, et al Education modifies the relation of AD pathology to level of cognitive function in older persons. Neurology. 2003;60(12):1909–15. 12821732 10.1212/01.wnl.0000069923.64550.9f

[6] Members ECC, Brayne C, Ince PG, Keage HA, McKeith IG, Matthews FE, et al Education, the brain and dementia: neuroprotection or compensation? Brain. 2010;133(Pt 8):2210–6. doi: 10.1093/brain/awq185 . 20826429 10.1093/brain/awq185

[7] Brickman AM, Siedlecki KL, Muraskin J, Manly JJ, Luchsinger JA, Yeung LK, et al White matter hyperintensities and cognition: Testing the reserve hypothesis. Neurobiol Aging. 2009; doi: 10.1016/j.neurobiolaging.2009.10.013 19926168 10.1016/j.neurobiolaging.2009.10.013 PMC2891625

[8] Beydoun MA, Beydoun HA, Gamaldo AA, Teel A, Zonderman AB, Wang Y. Epidemiologic studies of modifiable factors associated with cognition and dementia: systematic review and meta-analysis. BMC Public Health. 2014;14:643 doi: 10.1186/1471-2458-14-643 . 24962204 10.1186/1471-2458-14-643 PMC4099157

[9] Meng X, D'Arcy C. Education and dementia in the context of the cognitive reserve hypothesis: a systematic review with meta-analyses and qualitative analyses. PLoS ONE. 2012;7(6):e38268 doi: 10.1371/journal.pone.0038268 . 22675535 10.1371/journal.pone.0038268 PMC3366926

[10] Valenzuela MJ, Sachdev P. Brain reserve and dementia: a systematic review. Psychological Medicine. 2006;36(4):441–54. doi: 10.1017/S0033291705006264 16207391 10.1017/S0033291705006264

[11] Langa KM, Larson EB, Crimmins EM, Faul JD, Levine DA, Kabeto MU, et al A Comparison of the Prevalence of Dementia in the United States in 2000 and 2012. JAMA Intern Med. 2016 doi: 10.1001/jamainternmed.2016.6807 . 27893041 10.1001/jamainternmed.2016.6807 PMC5195883

[12] Chene G, Beiser A, Au R, Preis SR, Wolf PA, Dufouil C, et al Gender and incidence of dementia in the Framingham Heart Study from mid-adult life. Alzheimers Dement. 2015;11(3):310–20. doi: 10.1016/j.jalz.2013.10.005 24418058 10.1016/j.jalz.2013.10.005 PMC4092061

[13] Matthews FE, Arthur A, Barnes LE, Bond J, Jagger C, Robinson L, et al A two-decade comparison of prevalence of dementia in individuals aged 65 years and older from three geographical areas of England: results of the Cognitive Function and Ageing Study I and II. Lancet. 2013 doi: 10.1016/S0140-6736(13)61570-6 23871492 10.1016/S0140-6736(13)61570-6 PMC3906607

[14] Anstey K, Christensen H. Education, activity, health, blood pressure and apolipoprotein E as predictors of cognitive change in old age: a review. Gerontology. 2000;46(3):163–77. 10754375 10.1159/000022153

[15] Glymour MM, Weuve J, Berkman LF, Kawachi I, Robins JM. When is baseline adjustment useful in analyses of change? An example with education and cognitive change. Am J Epidemiol. 2005;162(3):267–78. doi: 10.1093/aje/kwi187 15987729 10.1093/aje/kwi187

[16] Karlamangla AS, Miller-Martinez D, Aneshensel CS, Seeman TE, Wight RG, Chodosh J. Trajectories of cognitive function in late life in the United States: demographic and socioeconomic predictors. Am J Epidemiol. 2009;170(3):331–42. doi: 10.1093/aje/kwp154 19605514 10.1093/aje/kwp154 PMC2727175

[17] Zahodne LB, Glymour MM, Sparks C, Bontempo D, Dixon RA, MacDonald SW, et al Education does not slow cognitive decline with aging: 12-year evidence from the victoria longitudinal study. J Int Neuropsychol Soc. 2011;17(6):1039–46. doi: 10.1017/S1355617711001044 . 21923980 10.1017/S1355617711001044 PMC3285821

[18] Muniz-Terrera G, Matthews F, Dening T, Huppert FA, Brayne C. Education and trajectories of cognitive decline over 9 years in very old people: methods and risk analysis. Age Ageing. 2009;38(3):277–82. doi: 10.1093/ageing/afp004 19252209 10.1093/ageing/afp004

[19] Schneider AL, Sharrett AR, Patel MD, Alonso A, Coresh J, Mosley T, et al Education and cognitive change over 15 years: the atherosclerosis risk in communities study. J Am Geriatr Soc. 2012;60(10):1847–53. doi: 10.1111/j.1532-5415.2012.04164.x . 23013064 10.1111/j.1532-5415.2012.04164.x PMC3662980

[20] Singh-Manoux A, Marmot MG, Glymour M, Sabia S, Kivimaki M, Dugravot A. Does cognitive reserve shape cognitive decline? Annals of Neurology. 2011;70(2):296–304. doi: 10.1002/ana.22391 21563209 10.1002/ana.22391 PMC3152621

[21] Marmot MG, Smith GD, Stansfeld S, Patel C, North F, Head J, et al Health inequalities among British civil servants: the Whitehall II study. Lancet. 1991;337(8754):1387–93. 1674771 10.1016/0140-6736(91)93068-k

[22] Singh-Manoux A, Kivimaki M, Glymour MM, Elbaz A, Berr C, Ebmeier KP, et al Timing of onset of cognitive decline: results from Whitehall II prospective cohort study. BMJ. 2012;344:d7622 doi: 10.1136/bmj.d7622 22223828 10.1136/bmj.d7622 PMC3281313

[23] Heim AW. AH 4 group test of general Intelligence. Windsor, UK: NFER-Nelson Publishing Company Ltd.; 1970 1970.

[24] Borkowski JG, Benton AL, Spreen O. Word fluency and brain damage. Neuropsychologica. 1967;5:135–40.

[25] Stansfeld SA, Marmot MG. Social class and minor psychiatric disorder in British Civil Servants: a validated screening survey using the General Health Questionnaire. Psychological Medicine. 1992;22(3):739–49. 1410098 10.1017/s0033291700038186

[26] Weuve J, Tchetgen Tchetgen EJ, Glymour MM, Beck TL, Aggarwal NT, Wilson RS, et al Accounting for bias due to selective attrition: the example of smoking and cognitive decline. Epidemiology. 2012;23(1):119–28. doi: 10.1097/EDE.0b013e318230e861 21989136 10.1097/EDE.0b013e318230e861 PMC3237815

[27] Fitzmaurice GM, Laird NM, Ware JH. Applied longitudinal analysis. Hoboken, New Jersey 2004 2004.

[28] Amieva H, Le GM, Millet X, Orgogozo JM, Peres K, Barberger-Gateau P, et al Prodromal Alzheimer's disease: Successive emergence of the clinical symptoms. Annals of Neurology. 2008;64(5):492–8. doi: 10.1002/ana.21509 19067364 10.1002/ana.21509

[29] Singh-Manoux A, Fayosse A, Sabia S, Canonico M, Bobak M, Elbaz A, et al Atrial fibrillation as a risk factor for cognitive decline and dementia. Eur Heart J. 2017 doi: 10.1093/eurheartj/ehx208 . 28460139 10.1093/eurheartj/ehx208 PMC5837240

[30] Case A, Paxson C. Height, Health, and Cognitive Function at Older Ages. Am Econ Rev. 2008;98(2):463–7. doi: 10.1257/aer.98.2.463 . 25152537 10.1257/aer.98.2.463 PMC4138508

[31] Sharp ES, Gatz M. Relationship between education and dementia: an updated systematic review. Alzheimer Dis Assoc Disord. 2011;25(4):289–304. doi: 10.1097/WAD.0b013e318211c83c . 21750453 10.1097/WAD.0b013e318211c83c PMC3193875

[32] Kozorovitskiy Y, Gross CG, Kopil C, Battaglia L, McBreen M, Stranahan AM, et al Experience induces structural and biochemical changes in the adult primate brain. Proc Natl Acad Sci U S A. 2005;102(48):17478–82. doi: 10.1073/pnas.0508817102 16299105 10.1073/pnas.0508817102 PMC1297690

[33] Markham JA, Greenough WT. Experience-driven brain plasticity: beyond the synapse. Neuron Glia Biol. 2004;1(4):351–63. doi: 10.1017/s1740925x05000219 16921405 10.1017/s1740925x05000219 PMC1550735

[34] West P. Rethinking the health selection explanation for health inequalities. Social Science & Medicine. 1991;32(4):373–84. doi: 10.1016/0277-9536(91)90338-D 2024152 10.1016/0277-9536(91)90338-d

[35] Gottesman RF, Rawlings AM, Sharrett AR, Albert M, Alonso A, Bandeen-Roche K, et al Impact of differential attrition on the association of education with cognitive change over 20 years of follow-up: the ARIC neurocognitive study. Am J Epidemiol. 2014;179(8):956–66. doi: 10.1093/aje/kwu020 . 24627572 10.1093/aje/kwu020 PMC3966720

[36] Barnett AG, van der Pols JC, Dobson AJ. Regression to the mean: what it is and how to deal with it. Int J Epidemiol. 2005;34(1):215–20. doi: 10.1093/ije/dyh299 15333621 10.1093/ije/dyh299

[37] Gatz M. Educating the brain to avoid dementia: can mental exercise prevent Alzheimer disease? PLoS Med. 2005;2(1):e7 doi: 10.1371/journal.pmed.0020007 . 15696217 10.1371/journal.pmed.0020007 PMC545200

[38] Lampit A, Hallock H, Valenzuela M. Computerized cognitive training in cognitively healthy older adults: a systematic review and meta-analysis of effect modifiers. PLoS Med. 2014;11(11):e1001756 doi: 10.1371/journal.pmed.1001756 . 25405755 10.1371/journal.pmed.1001756 PMC4236015

[39] Jack CR Jr., Knopman DS, Jagust WJ, Petersen RC, Weiner MW, Aisen PS, et al Tracking pathophysiological processes in Alzheimer's disease: an updated hypothetical model of dynamic biomarkers. Lancet Neurol. 2013;12(2):207–16. doi: 10.1016/S1474-4422(12)70291-0 23332364 10.1016/S1474-4422(12)70291-0 PMC3622225

[40] Villemagne VL, Burnham S, Bourgeat P, Brown B, Ellis KA, Salvado O, et al Amyloid beta deposition, neurodegeneration, and cognitive decline in sporadic Alzheimer's disease: a prospective cohort study. Lancet Neurol. 2013;12(4):357–67. doi: 10.1016/S1474-4422(13)70044-9 . 23477989 10.1016/S1474-4422(13)70044-9

[41] Batty GD, Shipley M, Tabak A, Singh-Manoux A, Brunner E, Britton A, et al Generalizability of occupational cohort study findings. Epidemiology. 2014;25(6):932–3. doi: 10.1097/EDE.0000000000000184 25265141 10.1097/EDE.0000000000000184

[42] Bennett DA, Schneider JA, Arvanitakis Z, Kelly JF, Aggarwal NT, Shah RC, et al Neuropathology of older persons without cognitive impairment from two community-based studies. Neurology. 2006;66(12):1837–44. doi: 10.1212/01.wnl.0000219668.47116.e6 . 16801647 10.1212/01.wnl.0000219668.47116.e6

[43] Hachinski V. Shifts in thinking about dementia. JAMA. 2008;300(18):2172–3. doi: 10.1001/jama.2008.525 19001628 10.1001/jama.2008.525

