# Supplementary material for: Contribution of cognitive performance and cognitive decline to associations between socioeconomic factors and dementia: A cohort study
Source: PLoS Med. 2017 Jun 26;14(6):e1002334. doi: 10.1371/journal.pmed.1002334 (PMC5484463; doi:10.1371/journal.pmed.1002334)
Supplement: S1 Table — (DOCX) [file pmed.1002334.s001.docx]

**S1 Table. Sample characteristics of participants included & not included in the analysis - overall and as a function of dementia status.^*^**

|  | **Participants included** | **Participants NOT included** | **p** |  | **Participants with dementia include** | **Participants with dementia NOT included** | **p** |
| --- | --- | --- | --- | --- | --- | --- | --- |
| **FROM BASELINE (1985)** | N=7,499 | N=2,439 |  |  | N=195 | N=134 |  |
| Female, % | 29.68 | 43.09 | <0.001 |  | 35.38 | 55.97 | <0.001 |
| Age, M(SD) | 44.58 (5.96) | 45.65 (6.18) | <0.001 |  | 50.74 (4.18) | 50.22 (4.85) | 0.30 |
| Non-white, % | 8.64 | 16.65 | <0.001 |  | 12.31 | 20.15 | 0.05 |
| Single, % | 23.82 | 30.96 | <0.001 |  | 23.08 | 35.82 | 0.01 |
| Height (metres), M(SD) | 1.72 (0.09) | 1.69 (0.10) | <0.001 |  | 1.71 (0.10) | 1.67 (0.11) | <0.001 |
| Low education, % | 44.27 | 56.42 | <0.001 |  | 54.87 | 67.91 | 0.05 |
| Low occupational position, % | 16.94 | 38.09 | <0.001 |  | 28.21 | 56.72 | <0.001 |
| Physically inactive**^†^**, % | 12.33 | 20.95 | <0.001 |  | 16.92 | 26.87 | 0.05 |
| Poor diet**^±^**, % | 40.02 | 46.17 | <0.001 |  | 37.44 | 46.97 | 0.10 |
| Heavy alcohol consumption**^‡^**, % | 16.34 | 14.02 | <0.001 |  | 16.41 | 8.27 | 0.002 |
| Current smokers, % | 15.00 | 27.06 | <0.001 |  | 14.87 | 29.10 | 0.007 |
| Systolic blood pressure, M(SD) | 122.30 (14.22) | 124.81 (15.07) | <0.001 |  | 124.75 (15.55) | 130.19 (15.74) | 0.002 |
| Diastolic blood pressure, M(SD) | 76.50 (9.94) | 77.89 (10.78) | <0.001 |  | 78.62 (10.04) | 81.42 (10.14) | 0.014 |
| Blood cholesterol, M(SD) | 5.91 (1.13) | 6.10 (1.25) | <0.001 |  | 6.28 (1.23) | 6.28 (1.19) | 0.951 |
| Body mass index, M(SD) | 24.48 (3.35) | 25.02 (3.97) | <0.001 |  | 25.53 (3.81) | 25.98 (4.29) | 0.317 |
| GHQ score, M(SD) | 3.68 (5.38) | 3.48 (5.59) | 0.11 |  | 3.74 (5.41) | 4.05 (6.16) | 0.63 |
| **CHRONIC CONDITIONS (1985-2015)** |  |  |  |  |  |  |  |
| COPD, % | 3.04 | 6.60 | <0.001 |  | 8.21 | 13.43 | 0.126 |
| Cancer, % | 11.42 | 13.05 | 0.031 |  | 13.85 | 14.93 | 0.783 |
| Diabetes, % | 8.09 | 12.01 | <0.001 |  | 15.38 | 29.10 | 0.003 |
| Dementia, % | 2.60 | 5.13 | <0.001 |  | - | - | - |
| Cardiovascular disease, % | 14.75 | 18.12 | <0.001 |  | 27.69 | 40.30 | 0.017 |

**^*^**The analyses are based on 195 dementia cases in 7,499 participants, weighted to reflect sample composed of 9,938 participants; please see Figure 1 for flow chart describing sample selection.

**^†^**Corresponds to <1hour/week moderately energetic and <1hour/week vigorous physical activity; **^±^**Corresponds to fruit and vegetable consumption <once a day; ‡Heavy alcohol consumption was defined as >14 units/week in women and > 21 units/week in men.

Abbreviations: M: Mean, SD: Standard deviation, COPD: Chronic Obstructive Pulmonary Disease, CVD: Cardiovascular Disease, including coronary heart disease and stroke, GHQ: General Health Questionnaire.
