# Supplementary material for: Contribution of cognitive performance and cognitive decline to associations between socioeconomic factors and dementia: A cohort study
Source: PLoS Med. 2017 Jun 26;14(6):e1002334. doi: 10.1371/journal.pmed.1002334 (PMC5484463; doi:10.1371/journal.pmed.1002334)
Supplement: S2 Table — (DOCX) [file pmed.1002334.s002.docx]

**S2 Table. Association of height, education, and occupation with performance and decline in the global cognitive score, excluding particpants with dementia.^†^**

|  | **COGNITIVE PERFORMANCE** | | |  | **15-YEAR COGNITIVE DECLINE** | | |
| --- | --- | --- | --- | --- | --- | --- | --- |
|  | **Mean (95% CI)** | **Difference (95% CI)** | **p** |  | **Mean (95% CI)** | **Difference (95% CI)** | **p** |
| **HEIGHT** |  |  |  |  |  |  |  |
| High | 0.07 (0.03, 0.11) | Ref. |  |  | -0.62 (-0.65, -0.59) | Ref. |  |
| Intermediate | -0.06 (-0.10, -0.02) | -0.13 (-0.19, -0.08) | <0.001 |  | -0.59 (-0.62, -0.56) | 0.03 (-0.01, 0.07) | 0.157 |
| Low | -0.15 (-0.19, -0.10) | -0.22 (-0.28, -0.16) | <0.001 |  | -0.62 (-0.65, -0.59) | 0.00 (-0.04, 0.05) | 0.858 |
| **EDUCATION** |  |  |  |  |  |  |  |
| High | 0.38 (0.35, 0.42) | Ref. |  |  | -0.63 (-0.67, -0.60) | Ref. |  |
| Intermediate | 0.04 (-0.00, 0.08) | -0.34 (-0.40, -0.29) | <0.001 |  | -0.62 (-0.65, -0.59) | 0.01 (-0.03, 0.06) | 0.580 |
| Low | -0.36 (-0.39, -0.32) | -0.74 (-0.79, -0.69) | <0.001 |  | -0.60 (-0.63, -0.57) | 0.03 (-0.01, 0.08) | 0.162 |
| **OCCUPATION** |  |  |  |  |  |  |  |
| High | 0.39 (0.36, 0.42) | Ref. |  |  | -0.63 (-0.66, -0.60) | Ref. |  |
| Intermediate | -0.17 (-0.20, -0.14) | -0.56 (-0.60, -0.51) | <0.001 |  | -0.62 (-0.65, -0.59) | 0.01 (-0.03, 0.05) | 0.544 |
| Low | -0.88 (-0.95, -0.81) | -1.27 (-1.35, -1.19) | <0.001 |  | -0.61 (-0.67, -0.55) | 0.02 (-0.05, 0.10) | 0.522 |

^†^Analysis based on inversely probability weighted generalized estimating equation (GEE) models, adjusted for age, sex, ethnicity, time-dependant marital status.
