# Supplementary material for: Contribution of cognitive performance and cognitive decline to associations between socioeconomic factors and dementia: A cohort study
Source: PLoS Med. 2017 Jun 26;14(6):e1002334. doi: 10.1371/journal.pmed.1002334 (PMC5484463; doi:10.1371/journal.pmed.1002334)
Supplement: S3 Table — (DOCX) [file pmed.1002334.s003.docx]

S3 Table. Association of height, education, and occupation with performance and decline in the global cognitive score: Adjustment for health behaviours and health status.^†^

|  | **COGNTIVE PERFORMANCE** | | |  | **15-YEAR COGNITIVE DECLINE** | | |
| --- | --- | --- | --- | --- | --- | --- | --- |
|  | **Mean (95% CI)** | **Difference (95% CI)** | **p** |  | **Mean (95% CI)** | **Difference (95% CI)** | **p** |
| **HEIGHT** |  |  |  |  |  |  |  |
| High | 0.04 (-0.00, 0.07) | Ref. |  |  | -0.60 (-0.63, -0.56) | Ref. |  |
| Intermediate | -0.08 (-0.12, -0.04) | -0.12 (-0.17, -0.06) | <0.001 |  | -0.57 (-0.60, -0.53) | 0.03 (-0.01, 0.07) | 0.186 |
| Low | -0.16 (-0.20, -0.12) | -0.20 (-0.25, -0.14) | <0.001 |  | -0.59 (-0.63, -0.55) | 0.01 (-0.04, 0.05) | 0.819 |
| **EDUCATION** |  |  |  |  |  |  |  |
| High | 0.35 (0.31, 0.38) | Ref. |  |  | -0.62 (-0.65, -0.58) | Ref. |  |
| Intermediate | 0.01 (-0.03, 0.06) | -0.33 (-0.39, -0.28) | <0.001 |  | -0.60 (-0.63, -0.56) | 0.02 (-0.03, 0.07) | 0.390 |
| Low | -0.37 (-0.40, -0.33) | -0.71 (-0.76, -0.66) | <0.001 |  | -0.57 (-0.60, -0.54) | 0.05 (-0.00, 0.09) | 0.053 |
| **OCCUPATION** |  |  |  |  |  |  |  |
| High | 0.36 (0.33, 0.39) | Ref. |  |  | -0.62 (-0.65, -0.58) | Ref. |  |
| Intermediate | -0.18 (-0.21, -0.15) | -0.54 (-0.59, -0.50) | <0.001 |  | -0.60 (-0.63, -0.57) | 0.02 (-0.02, 0.06) | 0.417 |
| Low | -0.87 (-0.94, -0.80) | -1.23 (-1.31, -1.15) | <0.001 |  | -0.57 (-0.63, -0.50) | 0.05 (-0.02, 0.12) | 0.189 |

**^†^**Inversely probability weighted (IPW) generalized estimating equation (GEE) models adjusted for age, sex, ethnicity, time-dependant marital status, smoking, alcohol consumption, physical activity, fruit and vegetable consumption, hypertension, diabetes, use of medication for cardiovascular disease, anxiety and depression symptoms, cardiovascular disease, chronic obstructive pulmonary disease.
