# Supplementary material for: Contribution of cognitive performance and cognitive decline to associations between socioeconomic factors and dementia: A cohort study
Source: PLoS Med. 2017 Jun 26;14(6):e1002334. doi: 10.1371/journal.pmed.1002334 (PMC5484463; doi:10.1371/journal.pmed.1002334)
Supplement: S4 Table — (DOCX) [file pmed.1002334.s004.docx]

**S4 Table. Sensitivity analysis: Association of socioeconomic markers with cognitive performance; Missing Not at Random (MNAR) assumption.**

|  | **Main analysis (Table 2)** | |  | **Sensitivity Analysis** | | | | | | | |
| --- | --- | --- | --- | --- | --- | --- | --- | --- | --- | --- | --- |
|  | **Estimates from weighted GEE** | |  | **Estimates from weighted GEE with Multiple Imputation** | |  | **Estimates from weighted GEE with Multiple Imputation**  **scenario 1^†^** | |  | **Estimates from weighted GEE with Multiple Imputation**  **scenario 2^†^** | |
|  | **Difference (95% CI)** | **p** |  | **Difference (95% CI)** | **p** |  | **Difference (95% CI)** | **p** |  | **Difference (95% CI)** | **p** |
| **HEIGHT** |  |  |  |  |  |  |  |  |  |  |  |
| High | Ref. |  |  | Ref. |  |  | Ref. |  |  | Ref. |  |
| Intermediate | -0.124 (-0.179, -0.069) | <0.001 |  | -0.109 (-0.161, -0.057) | <0.001 |  | -0.109 (-0.162, -0.056) | <0.001 |  | -0.109 (-0.163, -0.054) | <0.001 |
| Low | -0.213 (-0.271, -0.155) | <0.001 |  | -0.212 (-0.268, -0.156) | <0.001 |  | -0.216 (-0.273, -0.159) | <0.001 |  | -0.221 (-0.280, -0.162) | <0.001 |
| **EDUCATION** |  |  |  |  |  |  |  |  |  |  |  |
| High | Ref. |  |  | Ref. |  |  | Ref. |  |  | Ref. |  |
| Intermediate | -0.347 (-0.403, -0.291) | <0.001 |  | -0.363 (-0.417, -0.308) | <0.001 |  | -0.370 (-0.425, -0.315) | <0.001 |  | -0.380 (-0.437, -0.323) | <0.001 |
| Low | -0.746 (-0.798, -0.694) | <0.001 |  | -0.761 (-0.813, -0.710) | <0.001 |  | -0.768 (-0.821, -0.716) | <0.001 |  | -0.778 (-0.832, -0.724) | <0.001 |
| **OCCUPATION** |  |  |  |  |  |  |  |  |  |  |  |
| High | Ref. |  |  | Ref. |  |  | Ref. |  |  | Ref. |  |
| Intermediate | -0.565 (-0.609, -0.520) | <0.001 |  | -0.566 (-0.609, -0.524) | <0.001 |  | -0.570 (-0.613, -0.527) | <0.001 |  | -0.576 (-0.620, -0.531) | <0.001 |
| Low | -1.282 (-1.363, -1.201) | <0.001 |  | -1.309 (-1.386, -1.233) | <0.001 |  | -1.324 (-1.402, -1.247) | <0.001 |  | -1.346 (-1.426, -1.266) | <0.001 |

**^†^**Scenario 1 assumes that those with missing data have a cognitive score 0.2 SD below their imputed score, scenario 2 assumes this to be 0.5 SD lower.

GEE: Generalized Estimating Equations.
