# Supplementary material for: Contribution of cognitive performance and cognitive decline to associations between socioeconomic factors and dementia: A cohort study
Source: PLoS Med. 2017 Jun 26;14(6):e1002334. doi: 10.1371/journal.pmed.1002334 (PMC5484463; doi:10.1371/journal.pmed.1002334)
Supplement: S5 Table — (DOCX) [file pmed.1002334.s005.docx]

**S5 Table. Sensitivity analysis: Association of socioeconomic markers with cognitive decline; Missing Not at Random (MNAR) assumption.**

|  | **Main analysis (Table 2)** | |  |  | |  | **Sensitivity Analysis** | |  |  | |
| --- | --- | --- | --- | --- | --- | --- | --- | --- | --- | --- | --- |
|  | **Estimates from weighted GEE** | |  | **Estimates from weighted GEE with Multiple Imputation** | |  | **Estimates from weighted GEE with Multiple Imputation**  **scenario 1^†^** | |  | **Estimates from weighted GEE with Multiple Imputation**  **scenario 2^†^** | |
|  | **Difference (95% CI)** | **p** |  | **Difference (95% CI)** | **p** |  | **Difference (95% CI)** | **p** |  | **Difference (95% CI)** | **p** |
| **HEIGHT** |  |  |  |  |  |  |  |  |  |  |  |
| High | Ref. |  |  | Ref. |  |  | Ref. |  |  | Ref. |  |
| Intermediate | 0.024 (-0.020, 0.068) | 0.286 |  | 0.015 (-0.028, 0.057) | 0.500 |  | 0.014 (-0.030, 0.057) | 0.542 |  | 0.012 (-0.035, 0.059) | 0.623 |
| Low | 0.001 (-0.046, 0.048) | 0.967 |  | -0.011 (-0.059, 0.037) | 0.660 |  | -0.007 (-0.055, 0.042) | 0.792 |  | 0.000 (-0.053, 0.052) | 0.988 |
| **EDUCATION** |  |  |  |  |  |  |  |  |  |  |  |
| High | Ref. |  |  | Ref. |  |  | Ref. |  |  | Ref. |  |
| Intermediate | 0.022 (-0.027, 0.070) | 0.384 |  | 0.026 (-0.019, 0.072) | 0.253 |  | 0.029 (-0.018, 0.075) | 0.226 |  | 0.032 (-0.018, 0.082) | 0.213 |
| Low | 0.042 (-0.004, 0.088) | 0.073 |  | 0.050 (0.006, 0.094) | 0.027 |  | 0.050 (0.005, 0.094) | 0.030 |  | 0.049 (0.001, 0.097) | 0.046 |
| **OCCUPATION** |  |  |  |  |  |  |  |  |  |  |  |
| High | Ref. |  |  | Ref. |  |  | Ref. |  |  | Ref. |  |
| Intermediate | 0.013 (-0.027, 0.053) | 0.524 |  | 0.023 (-0.017, 0.064) | 0.259 |  | 0.024 (-0.017, 0.065) | 0.254 |  | 0.025 (-0.019, 0.069) | 0.269 |
| Low | 0.037 (-0.034, 0.109) | 0.307 |  | 0.048 (-0.026, 0.121) | 0.202 |  | 0.042 (-0.032, 0.117) | 0.263 |  | 0.034 (-0.046, 0.113) | 0.405 |

**^†^**Scenario 1 assumes that those with missing data have a cognitive score 0.2 SD below their imputed score, scenario 2 assumes this to be 0.5 SD lower.

GEE: Generalized Estimating Equations.
