# Supplementary material for: Contribution of cognitive performance and cognitive decline to associations between socioeconomic factors and dementia: A cohort study
Source: PLoS Med. 2017 Jun 26;14(6):e1002334. doi: 10.1371/journal.pmed.1002334 (PMC5484463; doi:10.1371/journal.pmed.1002334)
Supplement: S6 Table — (DOCX) [file pmed.1002334.s006.docx]

S6 Table. Association of height, education, and occupation with dementia: Unadjusted analyses.

|  | **N Dementia / Total = 320/9,938** | |
| --- | --- | --- |
| **N Dementia / N total** | | **Unadjusted** |
|  |  | **HR (95% CI)** |
| **HEIGHT** |  |  |
| High | 89/3261 | Ref. |
| Intermediate | 103/3364 | 1.13 (0.85, 1.49) |
| Low | 128/3313 | 1.46 (1.11, 1.91) |
| **EDUCATION** |  |  |
| High | 61/2603 | Ref. |
| Intermediate | 65/2639 | 1.07 (0.76, 1.52) |
| Low | 194/4696 | 1.85 (1.39, 2.46) |
| **OCCUPATION** |  |  |
| High | 89/3705 | Ref. |
| Intermediate | 111/4319 | 1.08 (0.82, 1.43) |
| Low | 120/1914 | 2.77 (2.11, 3.65) |
|  |  | 1.08 (0.82, 1.43) |
