# Supplementary material for: Contribution of cognitive performance and cognitive decline to associations between socioeconomic factors and dementia: A cohort study
Source: PLoS Med. 2017 Jun 26;14(6):e1002334. doi: 10.1371/journal.pmed.1002334 (PMC5484463; doi:10.1371/journal.pmed.1002334)
Supplement: S7 Table — (DOCX) [file pmed.1002334.s007.docx]

S7 Table. Association of cognitive performance and cognitive decline with dementia.

| **Dementia study population N Dementia / Total = 195/7,499** | **HR (95% CI)** |
| --- | --- |
| Cognitive performance (1SD higher) | 0.55 (0.47, 0.65) |
| Cognitive decline (1SD slower) | 0.80 (0.70, 0.90) |

SD: Standard Deviation

Cox model with age as time scale and adjusted for demographic characteristics (sex, ethnicity and time-dependant marital status) and 5 year birth cohort.
