# Supplementary material for: Contribution of cognitive performance and cognitive decline to associations between socioeconomic factors and dementia: A cohort study
Source: PLoS Med. 2017 Jun 26;14(6):e1002334. doi: 10.1371/journal.pmed.1002334 (PMC5484463; doi:10.1371/journal.pmed.1002334)
Supplement: S8 Table — (DOCX) [file pmed.1002334.s008.docx]

**S8 Table. Difference in cognitive trajectories prior to dementia by socioeconomic indicators**

|  | **Height** | |  | **Education** | |  | **Occupational position** | |
| --- | --- | --- | --- | --- | --- | --- | --- | --- |
| **Year** | Difference between intermediate and high | Difference between low and high |  | Difference between intermediate and high | Difference between low and high |  | Difference between intermediate and high | Difference between low and high |
| -18 | 0.37 | 0.08 |  | -0.12 | -0.82** |  | -0.96*** | -1.61*** |
| -17 | 0.37 | 0.06 |  | -0.21 | -0.87*** |  | -0.94*** | -1.62*** |
| -16 | 0.37 | 0.04 |  | -0.30 | -0.91*** |  | -0.92*** | -1.62*** |
| -15 | 0.36 | 0.03 |  | -0.37 | -0.93*** |  | -0.89*** | -1.61*** |
| -14 | 0.34 | 0.01 |  | -0.42* | -0.95*** |  | -0.86*** | -1.59*** |
| -13 | 0.33 | 0.00 |  | -0.47* | -0.95*** |  | -0.82*** | -1.56*** |
| -12 | 0.31 | -0.02 |  | -0.50** | -0.94*** |  | -0.79*** | -1.51*** |
| -11 | 0.29 | -0.03 |  | -0.51** | -0.93*** |  | -0.74*** | -1.46*** |
| -10 | 0.26 | -0.04 |  | -0.52** | -0.90*** |  | -0.69*** | -1.39*** |
| -9 | 0.23 | -0.05 |  | -0.51** | -0.86*** |  | -0.64*** | -1.32*** |
| -8 | 0.20 | -0.06 |  | -0.48** | -0.80*** |  | -0.59*** | -1.23*** |
| -7 | 0.17 | -0.07 |  | -0.45* | -0.74*** |  | -0.53*** | -1.14*** |
| -6 | 0.13 | -0.08 |  | -0.39* | -0.67*** |  | -0.47** | -1.03*** |
| -5 | 0.09 | -0.09 |  | -0.33 | -0.58*** |  | -0.40** | -0.92*** |
| -4 | 0.05 | -0.10 |  | -0.25 | -0.48** |  | -0.33* | -0.79*** |
| -3 | 0.00 | -0.10 |  | -0.16 | -0.38* |  | -0.25 | -0.65** |
| -2 | -0.05 | -0.11 |  | -0.06 | -0.26 |  | -0.17 | -0.50 |
| -1 | -0.10 | -0.11 |  | 0.06 | -0.13 |  | -0.09 | -0.35 |
| 0 | -0.16 | -0.12 |  | 0.19 | 0.02 |  | 0.00 | -0.18 |

*P<0.05, **P<0.01, ***P<0.001
