# Supplementary material for: Contribution of cognitive performance and cognitive decline to associations between socioeconomic factors and dementia: A cohort study
Source: PLoS Med. 2017 Jun 26;14(6):e1002334. doi: 10.1371/journal.pmed.1002334 (PMC5484463; doi:10.1371/journal.pmed.1002334)
Supplement: S1 Text — (DOCX) [file pmed.1002334.s009.docx]

**Prospective analysis plan**

The Whitehall II Study was established in 1985 to study social determinants in health and has involved seven clinical assessments so far. Before each clinical assessment we seek clearance from ethical governance bodies but at this stage in the study these are “substantial amendments” rather than a full application. We provide extracts from this application below.


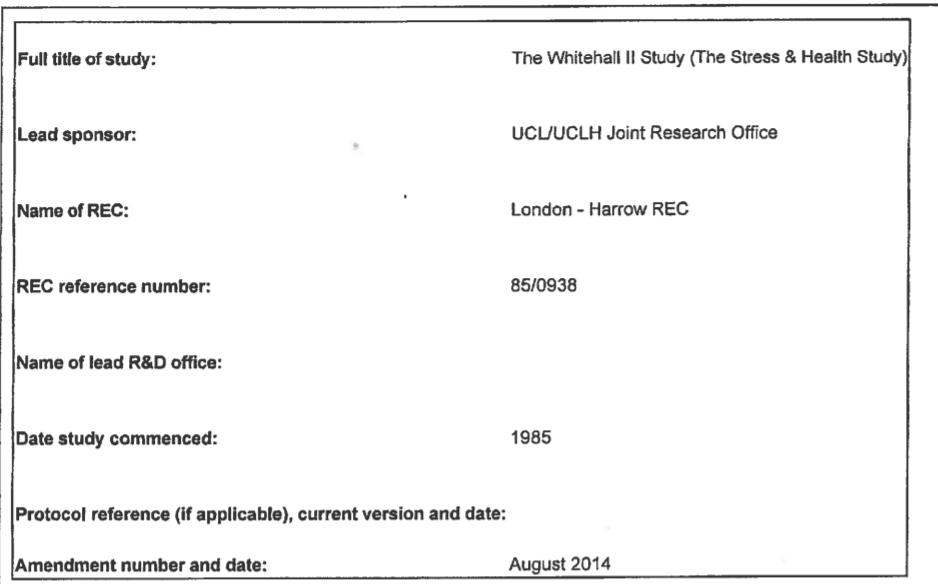


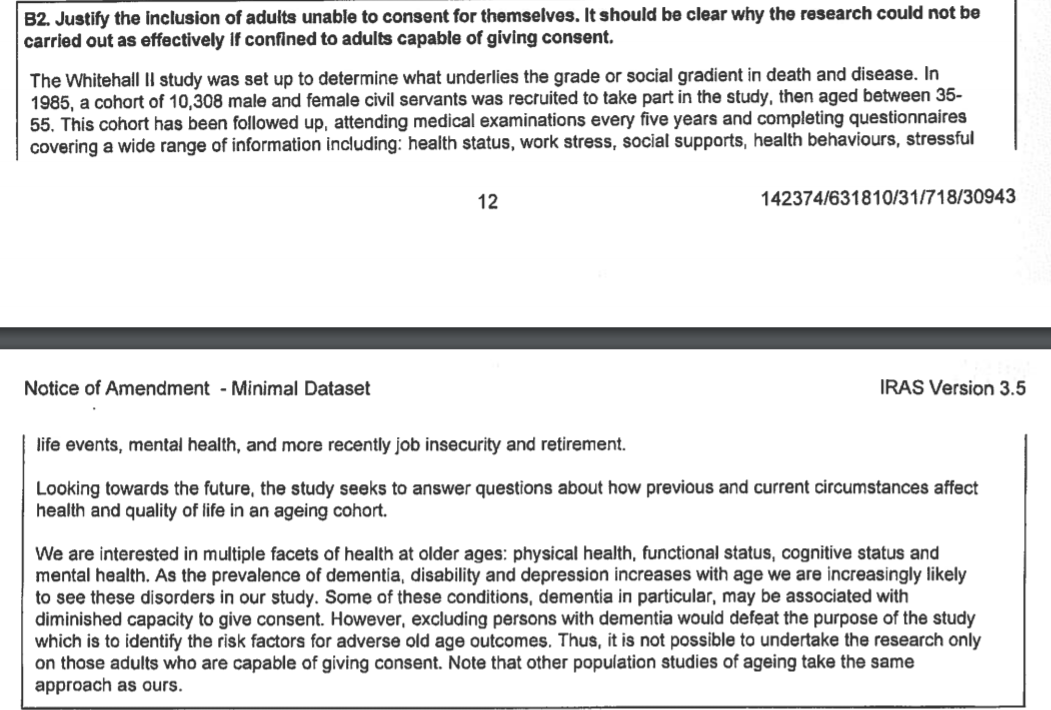


**Data analysis timeline**

May 2011: Publication of our results on cognitive reserve, showing markers of reserve to be associated with cognitive function but not cognitive decline.

- Singh-Manoux A, Marmot MG, Glymour M, Sabia S, Kivimaki M, Dugravot A. Does cognitive reserve shape cognitive decline? Annals of Neurology. 2011; 70: 296-304.

March 2014: Data from the 2012-2013 wave, including a fourth wave of cognitive data, become available for analysis.

March 2016: Data from linkage to electronic health records until 31/03/2015 retrieved and processed.

March –December 2016: Statistical analysis to test the cognitive reserve hypothesis. The analysis of cognitive performance and cognitive decline was undertaken using linear mixed effects model (LME), like in our 2011 paper. We used Cox regression for analysis of dementia. Both sets of analyses reflect standard epidemiological methods in longitudinal data. The Cox regression used Inverse Probability Weighting (IPW) to take missing data into account.

January 2017: Submission to PLOS Medicine

March-April 2017: In response to PLOS Medicine reviewers, we made two changes to the statistical analysis.

1. Missing data: The reviewers suggested that we use IPW for the analysis of cognitive decline. We reanalysed the data using weighted generalized estimating equations (GEE) linear regression model. This is an improvement on LME because 1) it allowed us to use IPW, and 2) unlike LME it does involves implicit imputation of cognitive scores beyond death.
2. Missing Not at Random (MNAR) The IPW method we use in the main analysis makes the Missing at Random (MAR) assumption (i.e. conditional on variables taken into account in the IPW, missing data do not depend on other unobserved variables). However, this assumption is untestable and as suggested by the reviewers we undertook sensitivity analyses with the MNAR assumption.
