# Supplementary material for: Contribution of cognitive performance and cognitive decline to associations between socioeconomic factors and dementia: A cohort study
Source: PLoS Med. 2017 Jun 26;14(6):e1002334. doi: 10.1371/journal.pmed.1002334 (PMC5484463; doi:10.1371/journal.pmed.1002334)
Supplement: S2 Text — (DOCX) [file pmed.1002334.s010.docx]

**Strengthening the Reporting of Observational Studies in Epidemiology (STROBE) Statement—Checklist of items that should be included in reports of observational studies.**

|  | Item No | Recommendation |
| --- | --- | --- |
| **Title and abstract** | 1 | 1. Indicate the study’s design with a commonly used term in the title or the abstract   **This study used a longitudinal cohort as indicated in the title.**  **“Contribution of cognitive performance and cognitive decline to associations between socioeconomic factors and dementia: a cohort study”** |
|  |  | 1. Provide in the abstract an informative and balanced summary of what was done and what was found   **The abstract contains the following sections: background, methods & findings, and conclusions.** |
| Introduction | | |
| Background/rationale | 2 | Explain the scientific background and rationale for the investigation being reported  **This is outlined in paragraphs 1 & 2 of the introduction.** |
| Objectives | 3 | State specific objectives, including any prespecified hypotheses  **This is outlined in paragraphs 3 & 4 of the introduction.** |
| Methods | | |
| Study design | 4 | Present key elements of study design early in the paper  **The abstract contains all the key elements of the paper.** |
| Setting | 5 | Describe the setting, locations, and relevant dates, including periods of recruitment, exposure, follow-up, and data collection  **This information is provided in the Methods section entitled “Study Design and participants”.** |
| Participants | 6 | (*a*) *Cohort study*—Give the eligibility criteria, and the sources and methods of selection of participants. Describe methods of follow-up  **This information is provided in the Methods section entitled “Study Design and participants”**  **“The Whitehall II study is an ongoing cohort study of men and women on 10,308 persons (6,895 men and 3,413 women) working in British Civil Service departments, aged 35-55 years, recruited to the study in 1985.[21] All participants responded to a questionnaire and underwent a structured clinical evaluation, consisting of measures of anthropometry, cardiovascular and metabolic risk factors and disease. Since the baseline medical examination, follow-up examinations have taken place approximately every 5 years (flow chart, Fig 1).”** |
|  |  |  |
| Variables | 7 | Clearly define all outcomes, exposures, predictors, potential confounders, and effect modifiers. Give diagnostic criteria, if applicable  **This information is described in the methods section under the heading “Measures” with the following subheadings**   - **Socioeconomic factors (1997)** - **Global cognitive score** - **Dementia** - **Covariates: Sociodemographic factors, health behaviours, health status** |
| Data sources/ measurement | 8* | For each variable of interest, give sources of data and details of methods of assessment (measurement). Describe comparability of assessment methods if there is more than one group  **This information is described in the methods section under the heading “Measures”.** |
| Bias | 9 | Describe any efforts to address potential sources of bias  **Bias due to missing data was addressed using Inverse Probability Weighting, described in the “Statistical Analysis” section, as follows.**  **“Analyses were weighted to take into account missing data using inverse-probability-weighting (IPW).[26] In order to do this, we used data on 9,938 participants who in 1997 (start of cognitive testing) were alive, non-demented, and had data on socioeconomic markers. The probability of remaining in the study sample was estimated using data on sociodemographic measures (age, sex, ethnicity, education, height, occupational position, marital status), health behaviours (smoking, alcohol consumption, physical activity, fruit and vegetable consumption), cardiometabolic risk factors (body mass index, systolic and diastolic blood pressure, cholesterol), and mental health (General Health Questionnaire) from study baseline (1985) and chronic conditions (hypertension, CHD, stroke, diabetes, chronic obstructive pulmonary disease, cancer) including dementia status over the follow-up (1985 to 2015). We also included interaction terms between dementia status and height, education, and occupation in the calculation of weights. The inverse of these probabilities were used to weight the analyses.”** |
| Study size | 10 | Explain how the study size was arrived at  **Fig 1 provides details of how we arrived at the sample size.** |
| Quantitative variables | 11 | Explain how quantitative variables were handled in the analyses. If applicable, describe which groupings were chosen and why  **This is described in the Methods section.** |
| Statistical methods | 12 | (*a*) Describe all statistical methods, including those used to control for confounding **The statistical methods are described on page 9 to 11 and all confounders are described under the section covariates.** |
|  |  | 1. Describe any methods used to examine subgroups and interactions   **We examined sex differences, described as follows in the manuscript:**  **“As there was no evidence of sex difference (p for interaction for height=0.09, education=0.40, and occupation=0.86) men and women were combined in Cox regression, with age as the time scale.”** |
|  |  | 1. Explain how missing data were addressed   **We used Inverse Probability Weighting to take missing data into account, described in paragraph 2 of the statistical methods section.** |
|  |  | 1. *Cohort study*—If applicable, explain how loss to follow-up was addressed   **Please see section on sensitivity analyses below.** |
|  |  | 1. Describe any sensitivity analyses   **We undertook sensitivity analyses with the missing not at random (MNAR) assumption where we tested two scenarios; described on paragraph four of the statistical analysis section as below.**  **“Sensitivity analysis: Our main analysis using weighted GEE is based on a missing at random (MAR) assumption, i.e., given the variables used to define weights, data are missing independently of unobserved data. As this assumption is untestable, we undertook sensitivity analyses under a missing not at random (MNAR) assumption following three steps. First, we used multiple imputation (20 datasets) to impute missing cognitive data based on covariates used in IPW, and all cognitive data, measures of functional status using the SF36-physical and mental component scores, and instrumental and basic activities of daily living (IADL & ADL) over the follow-up (1997 to 2012); data imputed after death were set as missing. Second, we replaced the imputed cognitive data by values that were 0.2 or 0.5 Standard Deviations (SD) lower, i.e., those who had missing cognitive data are hypothesized to systematically have lower scores than participants with the same characteristics for whom cognitive data were available. Third, we repeated the weighted GEE analysis on the imputed dataset using Rubin’s rule to compute estimates.”**  **Please see results in S4 Table and S5 Table.** |

| Results | | |
| --- | --- | --- |
| Participants | 13* | 1. Report numbers of individuals at each stage of study—eg numbers potentially eligible, examined for eligibility, confirmed eligible, included in the study, completing follow-up, and analysed   **Fig 1 provides details on the composition of the analytic sample.** |
|  |  | 1. Give reasons for non-participation at each stage   **S1 Table described the characteristics of participants included in the analysis compared to those not included.** |
|  |  | 1. Consider use of a flow diagram   **A flow diagram is included as Fig 1.** |
| Descriptive data | 14* | 1. Give characteristics of study participants (eg demographic, clinical, social) and information on exposures and potential confounders   **These characteristics are included in Table 1 and S1 Table.** |
|  |  | 1. Indicate number of participants with missing data for each variable of interest   **These data are provided in Fig 1.** |
|  |  | 1. *Cohort study*—Summarise follow-up time (eg, average and total amount)   **Figure 1 provides details of the follow-up; follow-up time is described in the manuscript as follows:**  **“One dementia case occurred before 1997, and covariate data were missing for 8 cases, leaving 320 cases in the analysis over a mean follow-up of 16.1 years.”** |
| Outcome data | 15* | *Cohort study*—Report numbers of outcome events or summary measures over time  **The outcome numbers are described in the abstract, results section and Fig 1.** |
|  |  | *Case-control study—*Report numbers in each exposure category, or summary measures of exposure  **Not relevant.** |
|  |  | *Cross-sectional study—*Report numbers of outcome events or summary measures  **Not relevant.** |
| Main results | 16 | 1. Give unadjusted estimates and, if applicable, confounder-adjusted estimates and their precision (eg, 95% confidence interval). Make clear which confounders were adjusted for and why they were included   **Unadjusted estimates are provided in the supplementary data, S6 Table and S7 Table. Confounders and potential mediators are described in the methods section.** |
|  |  | (*b*) Report category boundaries when continuous variables were categorized  **For height we provide the following information.**  **“Tertiles of height were used; 174-179 cm (160-165 cm) in men (women) was the middle category and height above or below this range to constitute the other two categories.”** |
|  |  | 1. If relevant, consider translating estimates of relative risk into absolute risk for a meaningful time period   **Not relevant.** |
| Other analyses | 17 | Report other analyses done—eg analyses of subgroups and interactions, and sensitivity analyses  **The** **Supplementary data (S1 Table to S8 Table) provide details of analyses undertaken to complement those reported in the manuscript.**  **Sensitivity analyses are described as follows**  **“Sensitivity analysis: Our main analysis using weighted GEE is based on a missing at random (MAR) assumption, i.e., given the variables used to define weights, data are missing independently of unobserved data. As this assumption is untestable, we undertook sensitivity analyses under a missing not at random (MNAR) assumption following three steps. First, we used multiple imputation (20 datasets) to impute missing cognitive data based on covariates used in IPW, and all cognitive data, measures of functional status using the SF36-physical and mental component scores, and instrumental and basic activities of daily living (IADL & ADL) over the follow-up (1997 to 2012); data imputed after death were set as missing. Second, we replaced the imputed cognitive data by values that were 0.2 or 0.5 Standard Deviations (SD) lower, i.e., those who had missing cognitive data are hypothesized to systematically have lower scores than participants with the same characteristics for whom cognitive data were available. Third, we repeated the weighted GEE analysis on the imputed dataset using Rubin’s rule to compute estimates.”**  **Please see results in Table S4 and S5.** |
| Discussion | | |
| Key results | 18 | Summarise key results with reference to study objectives  **The first paragraph of the discussion section summarises results in relation to study objectives.** |
| Limitations | 19 | Discuss limitations of the study, taking into account sources of potential bias or imprecision. Discuss both direction and magnitude of any potential bias  **Limitations of the study are discussed in the penultimate paragraph of the discussion section.** |
| Interpretation | 20 | Give a cautious overall interpretation of results considering objectives, limitations, multiplicity of analyses, results from similar studies, and other relevant evidence  **The discussion section considers our results in light of previous findings in this domain.** |
| Generalisability | 21 | Discuss the generalisability (external validity) of the study results  **This is described in the discussion section, penultimate paragraph.** |
| Other information | | |
| Funding | 22 | Give the source of funding and the role of the funders for the present study and, if applicable, for the original study on which the present article is based  **Detailed in the additional submission required information, as follows.**  **The Whitehall II study is supported by grants from the US National Institutes on Aging (R01AG013196; R01AG034454); the UK Medical Research Council (MRC K013351) and British Heart Foundation. MK is supported by the UK Medical Research Council (K013351), NordForsk (75021), and Horizon2020 (Lifepath 633666). ASM and JPM are supported by Horizon2020 (Fresher 643357).** |

*Give information separately for cases and controls in case-control studies and, if applicable, for exposed and unexposed groups in cohort and cross-sectional studies.

**Note:** An Explanation and Elaboration article discusses each checklist item and gives methodological background and published examples of transparent reporting. The STROBE checklist is best used in conjunction with this article (freely available on the Web sites of PLoS Medicine at http://www.plosmedicine.org/, Annals of Internal Medicine at http://www.annals.org/, and Epidemiology at http://www.epidem.com/). Information on the STROBE Initiative is available at www.strobe-statement.org.
